# Performance evaluation of commercial library construction kits for PCR-based targeted sequencing using a unique molecular identifier

**DOI:** 10.1186/s12864-019-5583-7

**Published:** 2019-03-14

**Authors:** Jongsuk Chung, Ki-Wook Lee, Chung Lee, Seung-Ho Shin, Sungkyu Kyung, Hyo-Jeong Jeon, Sook-Young Kim, Eunjung Cho, Chang Eun Yoo, Dae-Soon Son, Woong-Yang Park, Donghyun Park

**Affiliations:** 1 Samsung Genome Institute, Samsung Medical Center, Seoul, 06351 South Korea; 2 Department of Molecular Cell Biology, Sungkyunkwan University School of Medicine, Suwon, 16419 South Korea; 3 Department of Digital Health, SAIHST, Sungkyunkwan University, Seoul, 06351 South Korea; 4 Department of Health Sciences and Technology, SAIHST, Sungkyunkwan University, Seoul, 06351 South Korea; 5 Department of Bioinformatics and Life Science, Soongsil University, Seoul, 06978 South Korea; 6 GENINUS Inc., Seoul, 05836 South Korea

**Keywords:** UMI, NGS, PCR-based, Efficiency, Comparison, Evaluation

## Abstract

**Background:**

Target enrichment is a critical component of targeted deep next-generation sequencing for the cost-effective and sensitive detection of mutations, which is predominantly performed by either hybrid selection or PCR. Despite the advantages of efficient enrichment, PCR-based methods preclude the identification of PCR duplicates and their subsequent removal. Recently, this limitation was overcome by assigning a unique molecular identifier(UMI) to each template molecule. Currently, several commercial library construction kits based on PCR enrichment are available for UMIs, but there have been no systematic studies to compare their performances. In this study, we evaluated and compared the performances of five commercial library kits from four vendors: the Archer® Reveal ctDNA™ 28 Kit, NEBNext Direct® Cancer HotSpot Panel, Nugen Ovation® Custom Target Enrichment System, Qiagen Human Comprehensive Cancer Panel(HCCP), and Qiagen Human Actionable Solid Tumor Panel(HASTP).

**Results:**

We evaluated and compared the performances of the five kits using 50 ng of genomic DNA for the library construction in terms of the library complexity, coverage uniformity, and errors in the UMIs. While the duplicate rates for all kits were dramatically decreased by identifying unique molecules with UMIs, the Qiagen HASTP achieved the highest library complexity based on the depth of unique coverage indicating superb library construction efficiency. Regarding the coverage uniformity, the kits from Nugen and NEB performed the best followed by the kits from Qiagen. We also analyzed the UMIs, including errors, which allowed us to adjust the depth of unique coverage and the length required for sufficient complexity. Based on these comparisons, we selected the Qiagen HASTP for further performance evaluations. The targeted deep sequencing method based on PCR target enrichment combined with UMI tagging sensitively detected mutations present at a frequency as low as 1% using 6.25 ng of human genomic DNA as the starting material.

**Conclusion:**

This study is the first systematic evaluation of commercial library construction kits for PCR-based targeted deep sequencing utilizing UMIs. Because the kits displayed significant variability in different quality metrics, our study offers a practical guideline for researchers to choose appropriate options for PCR-based targeted sequencing and useful benchmark data for evaluating new kits.

**Electronic supplementary material:**

The online version of this article (10.1186/s12864-019-5583-7) contains supplementary material, which is available to authorized users.

## Background

Cancer genome profiling by massively parallel sequencing has rapidly advanced our understanding of the molecular characteristics underlying tumorigenesis [1–3]. Furthermore, cataloging the most frequently mutated cancer genes across various cancer types [4, 5] has made targeted resequencing an attractive option to cost-effectively analyze genetic alterations in tumor specimens [6, 7]. Whereas whole genome sequencing (WGS) or whole exome sequencing (WES) provides additional information on genomic variants across broad regions of the human genome, [8, 9] targeted sequencing offers distinct advantages over these methods by reducing costs and simplifying data management/analysis. The advantages of targeted deep sequencing are particularly obvious in clinical settings where the selection of therapy is the primary reason for genomic profiling and only a small fraction of identified mutations are potentially responsive to a therapy (i.e., actionable mutations) [10, 11]. While the targeted sequencing method has been successfully employed for clinical genomic profiling, sufficient sequencing coverage was repeatedly suggested as a prerequisite for the successful implementation of the method in clinical cancer genome profiling [12, 13].

As we recently reported, significant proportions of clinically actionable variants have allele fractions as low as less than 5%, often because of low tumor purity, heterogeneity, and secondary tumor driver mutations resulting from treatment [14, 15]. While the detection of low variant allele fractions (VAFs) requires the sequencing of a sufficient number of molecules, low quantity and quality of DNA extracted from clinical tissue samples often pose obstacles. Clinical tissue specimens available for genetic profiling are often minute, which regularly consist of several sections of formalin-fixed paraffin embedded (FFPE) tissues or needle biopsy samples [16]. The technical challenge becomes even greater when cancer somatic mutations are interrogated from liquid biopsies such as plasma cell-free DNA (cfDNA) samples [17, 18]. The amount of cfDNA ranges from 1 to 100 ng/mL in the plasma [19], and the allele frequency of tumor DNA in cfDNA is very low, often lower than 1% [20]. The detection of low VAF variants in cfDNA samples is particularly challenging because high complexity sequencing libraries must be generated using a limited amount of input DNA. In this regard, it is critical to construct sequencing libraries with a high recovery rate of cfDNA molecules.

For target enrichment, hybrid selection-based capture [21] and PCR amplification [22] are two major techniques. In general, the hybrid selection-based capture method using biotinylated oligonucleotides complementary to target regions and streptavidin-coated magnetic beads is more expensive, involves more steps, and requires more input DNA than PCR-based methods. In contrast, capture-based enrichment methods are better suited for identifying unique molecules and efficiently removing PCR duplicates. Because capture-based enrichment typically uses input DNA generated by random fragmentation, PCR duplicates can be readily identifiable by the unique start and end positions of each fragment. In contrast, because PCR-based enrichment methods generate fragments with the same genomic positions defined by pairs of PCR primers, PCR duplicates and copies of unique molecules are virtually indistinguishable. Because of their inability to remove PCR duplicates, PCR-based enrichment methods are prone to false positives, particularly when calling low VAF variants. After distinct short oligonucleotides with random sequences tagging each template molecule were proposed as unique molecular identifiers (UMIs, also known as molecular barcodes), [23–26] accurately distinguishing PCR duplicates from copies of unique fragments generated by a pair of PCR primer became possible. Consequently, UMIs have reduced quantitative bias during experimental processes and, thus, can be adopted for the accurate quantification of target templates. Strategies using UMIs were also used to detect ultra-rare variants, as errors arising from artifacts during library construction and sequencing runs could be eliminated by comparing the sequences of PCR duplicates identified with a UMI sequence [27, 28]. PCR-based targeted sequencing utilizing UMIs became readily accessible to researchers, as several vendors such as ArcherDx, NEB, Nugen, and Qiagen developed and commercialized library construction kits. All kits use a target enrichment step involving PCR amplification and adopt UMIs to tag each template molecule, but the specific techniques creating library molecules differ among vendors. For example, the enrichment method of the NEBNext Direct Cancer Hotspot Panel involves a combination of hybrid section-based capture and PCR amplification in which a hybridized bait sequence is used as a primer to enrich targets that are distinct from the others. Adaptor ligation and subsequent PCR amplification using combinations of primers targeting specific genomic regions and a universal adaptor sequence are used in the ArcherDx and Qiagen kits. Nugen’s targeted enrichment method described as single primer enrichment by the manufacturer is also similar to the ArcherDx and Qiagen kits [29]. Each library construction kit differentially places their own UMI in the adaptors. As a result, the UMIs of the ArcherDx Reveal ctDNA 28 kit [30] are included in the read 1 fastq, while the UMIs of the Qiagen kits are in the read 2 fastq. In contrast, Nugen and NEB have UMIs in the sample index region. The features of each kit including variant calling and the method of UMI tagging and enrichment of each kit are summarized (Additional files 1, 2 and 3).

The performances of these commercial kits using a PCR-based target enrichment method incorporated with the UMI sequence have not been systematically compared yet. In this study, we examined five kits: the Archer® Reveal ctDNA™ 28 Kit, NEBNext Direct® Cancer HotSpot Panel, Nugen Ovation® Custom Target Enrichment System, Qiagen Human Comprehensive Cancer Panel, and Qiagen Human Actionable Solid Tumor Panel. The performance of each kit was evaluated and compared in terms of library construction efficiency, uniformity of the target region, and UMI sequence errors. Next, we selected the Qiagen Human Actionable Solid Tumor Panel kit and further evaluated its detection sensitivity to identify low VAF variants using a limited amount of genomic DNA in the range of 6.25–50 ng. Performance evaluations of these commercial kits offer valuable benchmark data for the future evaluation of PCR-based targeted sequencing methods.

## Results

### Evaluation of the library construction efficiencies of the commercial kits utilizing UMIs

We first compared the library construction efficiency of five commercial library kits exploiting UMI sequences. We used 50 ng of genomic DNA purified from HapMap cell lines for the library construction. Triplicated libraries of each kit were constructed according to the corresponding manufacturer’s protocol (Materials and Methods). Aiming to achieve a raw read depth of no less than 10,000×, we generated datasets (*n* = 3 for each kit) where the depth of coverage varied from 13,337× to 43,048×, except for the Qiagen HCCP (Additional file 1). Because its total target region (920 kb) is exceptionally large compared to the other kits (15–48.1kb), an average depth of coverage obtained using the Qiagen HCCP was 8148 × .

After aligning the raw reads to the reference genome hg19 using BWA-mem [31], deduplication based on the UMI sequences was performed using the fgbio package (https://github.com/fulcrumgenomics/fgbio) by comparing the UMI sequences of fragments (i.e., pairs of reads) with identical start/end genomic positions. In parallel, deduplication by the genomic positions of DNA fragments without using the UMI sequences was carried out by PICARD. For each primer, the depth of coverage was expected to be the highest at the position next to the 3′ end of the primer and to decrease as the position moved away from the 3′ end of the primer. Thus, we examined the mean depth by increasing the size of the target regions from 50 to 250 base pairs (bp) adjacent to the 3′ end of each primer, as the mean depth of unique coverage can vary depending on how large the regions defined as target regions were. As expected, the depth of coverage decreased as the target regions increased from the end of the primer, which was consistently observed in all kits tested (Additional file 4). However, we did not examine the NEB kit because the manufacturer had not made the genomic positions of the probes open to the public.

**Fig. 1:**
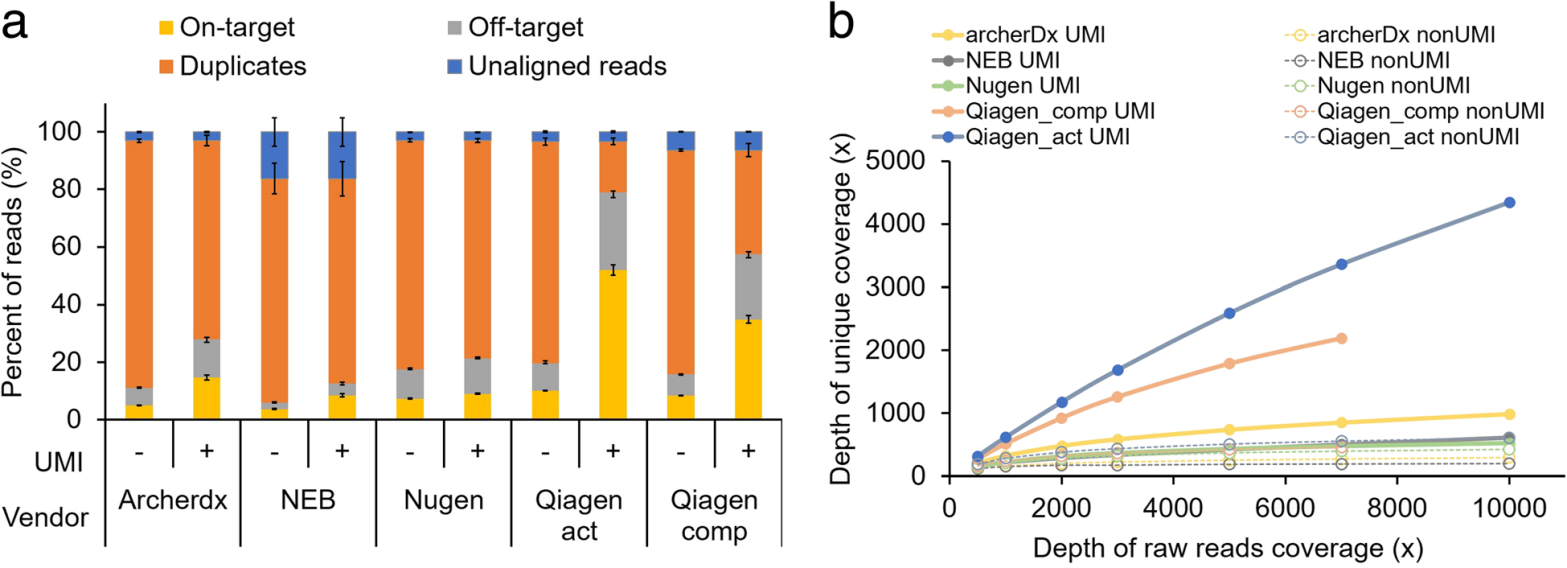
Comparison of kits using 50 ng of cell line genomic DNA. **a** Stacked bar plot showing the fractions of filtered reads (i.e., unaligned, duplicated, and off-target reads) and reads remaining after filtering (i.e., on-target) during raw data processing for five commercial kits with and without UMIs for deduplication. **b** Mean depth of unique coverage after filtering according to total raw read depth

To compare the rate of on-target reads among the different kits, we examined sequencing metrics by defining the target regions as the adjacent 100-bp region to the 3′ end of each primer (Fig. 1a). For accurate comparisons, fastq files were down-sampled to 5000× raw read coverage by randomly selecting reads. In a comparison of the sequencing metrics for the five kits, the Qiagen HASTP showed the highest on-target rate when UMIs were used to identify unique molecules and PCR duplicates (Fig. 1a). Without using UMIs, the Qiagen HASTP kit displayed only a 10.1% on-target rate when deduplication was performed based on the genomic positions of the fragments. However, when UMIs were used for deduplication, the on-target rate increased to 52.0% (Fig. 1a). All five kits showed on-target rate increases of 1.7–41.9% by identifying unique molecules with the UMIs, which concomitantly displayed a 1.19–5.13-fold higher mean depth of unique coverage compared to without the UMIs (Fig. 1). This UMI effect was primarily driven by the reduction in duplicate rates because the start/end positions of the fragments were not sufficiently complex to identify most unique molecules.

To estimate the library construction efficiency depending on the data size, we down-sampled each raw fastq data to various data sizes with 500 × − 10,000× coverage. We adjusted the data size based on the depth of raw read coverage (i.e., total bases divided by the sizes of the total target regions) rather than on the total read counts because the sizes of the total target regions were diverse across the five kits. As described above, we could not obtain 10,000× coverage data for the Qiagen HCCP. The depth of unique coverage based on deduplication without UMIs did not proportionally increase according to the total data size, particularly when the depth of raw read coverage was greater than a few thousand (Fig. 1b). However, coverage depth after deduplication using UMIs was not completely saturated at a 10,000× raw read coverage, indicating that further data generation can identify more unique molecules (Fig. 1b). Among the five commercial kits using UMIs, the Qiagen HASTP showed the highest mean depth of unique coverage followed by the Qiagen HCCP (Fig. 1b).

Next, we compared the uniformity of coverage depth across the target regions among the five kits. When we examined the distribution of the read depth of each kit using data sets adjusted to 5000× by in silico down-sampling, the depth distributions from the Nugen, NEB, and Qiagen HCCP kits were more uniform compared to the other kits (Additional file 5). In the Nugen kit, the percent of positions at which the depth of coverage was more than twice the average depth was 14.3% and the percent of positions at which the depth of coverage was less than a half of the average depth was 14.2%. These values from the NEB and Qiagen HCCP kits were similar to those of the Nugen kit. In contrast, the two observed values were relatively elevated in the Qiagen HASTP and even further in the ArcherDX, indicating a less uniform coverage depth than the other kits. The uniformity of the coverage depth was also visualized by plotting the coverage efficiency of the percentage of total targeted bases covered at specific depths, which consistently indicated the relative degree of uniformity of the depth distributions among the kits (Additional file 5).

### Errors in the UMIs

**Fig. 2:**
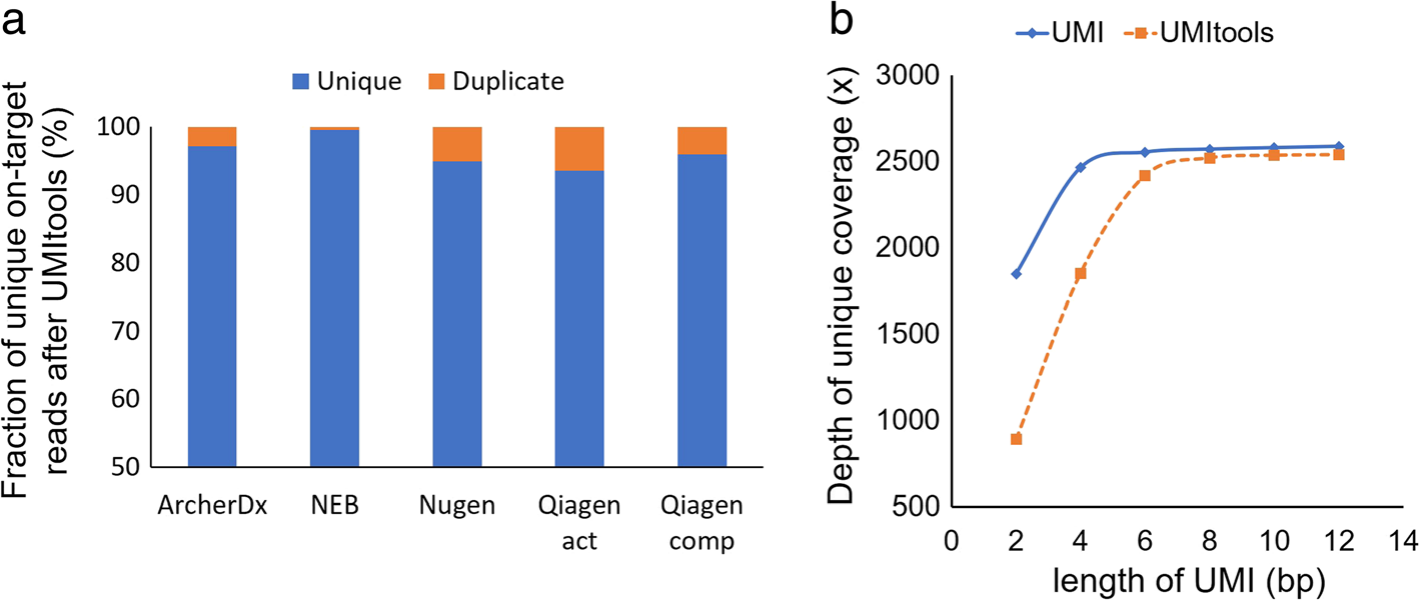
Analysis of UMIs. **a** Stacked bar plot showing the fractions of unique on-target reads that are authentic (i.e., unique) and inflated (duplicated) due to UMI errors. Estimation was performed using UMI-tools. **b** Mean depth of unique coverage according to the length of the UMI sequence

The identification of PCR duplicates by UMIs enables researchers to filter out random errors introduced during library construction and the sequencing run. However, these random errors arise not only in sequences from template fragments but also in UMI sequences. If errors occurred in the UMIs, then PCR duplicates would be mistaken for copies arising from unique molecules tagged by different UMIs, resulting in the overestimation of the depth of unique coverage. To estimate the levels of errors in the UMIs, we analyzed our data using UMI-tools, [32] which was developed to accurately identify unique molecules by accounting for UMI errors. The method infers errors based on the topologies of UMI sequence networks. Applying UMI-tools, we estimated the UMI error rates of the five kits to be 0.1–0.4% (Additional file 6), demonstrating that only a minor fraction of reads had the wrong UMI sequence due to sequencing errors. For all the kits, unique on-target read rates decreased after the UMI-tools process because duplicated reads with different UMI sequences due to errors were removed. While the result from NEB displayed the smallest decrease (0.04%), the unique on-target read rates were adjusted to less than 6.4% in the tested kits based on the UMI error estimations (Fig. 2a). Nevertheless, after correction, the Qiagen HASTP still showed the highest mean depth of unique coverage followed by the Qiagen HCCP (Additional file 6).

### Length of the UMIs

Next, we examined the most suitable length of the UMI sequence. Using raw data from the Qiagen HASTP, which contains 12-bp UMI sequences, the UMIs were cut to different lengths increasing from 2 to 12 bp in 2-bp steps, and deduplication was executed with each of the UMIs of different lengths. The results showed that the depth of unique coverage increased according to the length of the UMI from 2 to 6 bp (Fig. 2b). However, when the UMI length varied from 6 to 12 bp, there was no significant difference in the depth of unique coverage. These results indicated that 6 bp is the minimum length necessary to create sufficient complexity in the sequencing data at this depth level. When unique molecules were identified by UMI-tools, the minimum UMI length increased from 6 to 8 bp, which is consistent with the results of a previous study [32]. Because the lengths of the UMIs directly correlate with their diversity, the minimal required length may increase depending on the number of unique molecules. Because we estimated the UMI length with a data set generated using 50 ng of genomic DNA and down-sampled to 5000× coverage, the minimally required length would increase if more genomic DNA was used and/or if more data were generated.

### Performance of the selected library preparation method using PCR-based enrichment technology with UMI sequences

**Fig. 3:**
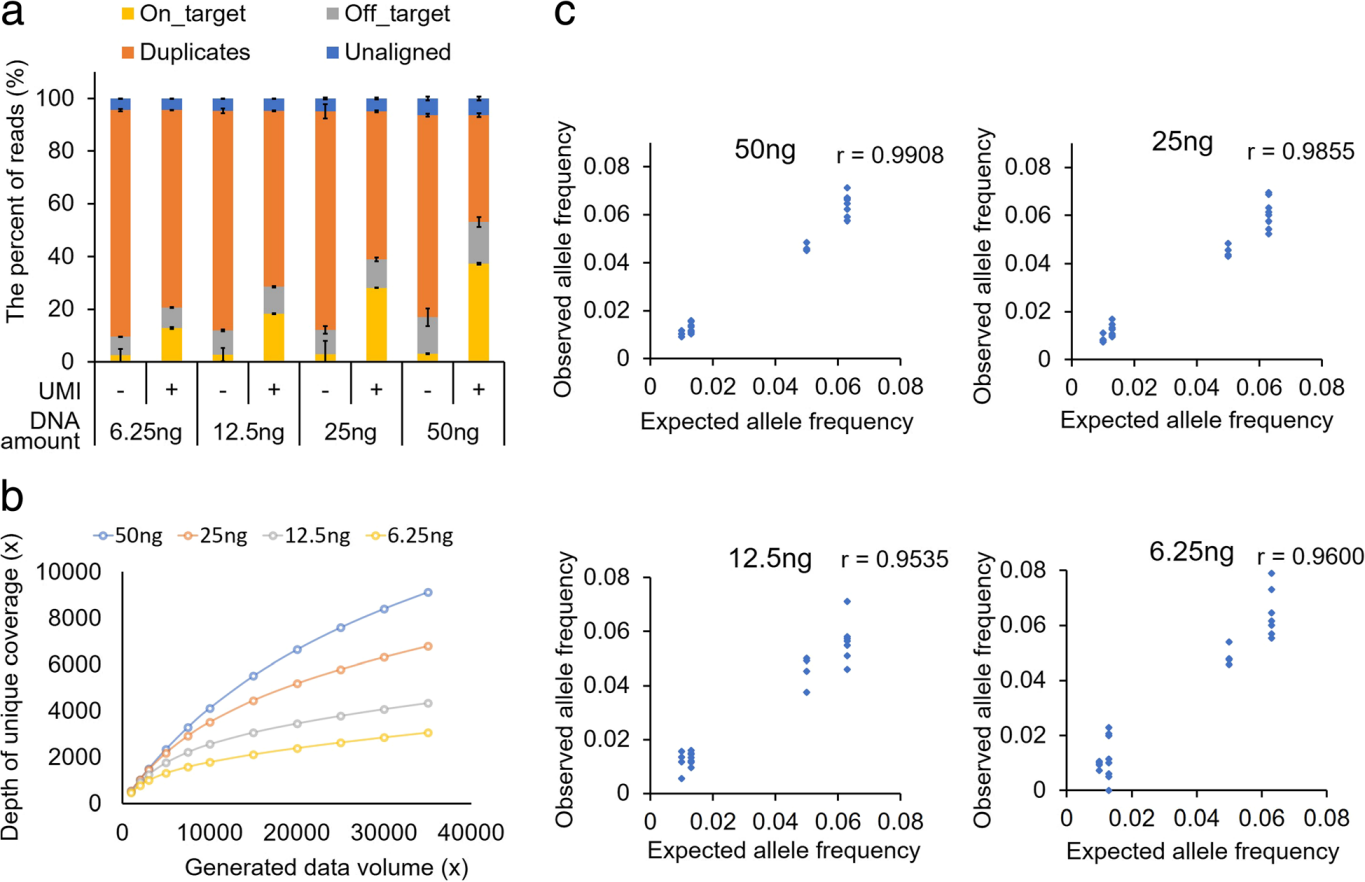
Performance evaluation of Qiagen HASTP kit. **a** Stacked bar plot showing the fractions of filtered reads (i.e., unaligned, duplicated, and off-target reads) and reads remaining after filtering (i.e., on-target) during raw data processing for five commercial kits with and without UMIs for deduplication. **b** Mean depth of unique coverage after filtering with UMIs according to the initial genomic DNA amount. **c** Correlation between expected allele frequencies of variants in reference material and observed allele frequencies of variants from Qiagen HASTP

After comparing the commercial kits, we selected the Qiagen HASTP and evaluated the analytical performance of the kit using varying amounts of genomic DNA (6.25, 12.5, 25, and 50 ng), which was technically duplicated to ensure repeatability. To evaluate the detection sensitivity for somatic mutations, we used the Horizon cfDNA reference standard set, which contains hotspot mutations at a frequency of 0.1–6.3%. On average, 17.1 million reads were generated from each sample; the number of reads varied between 12.2 and 22.9 million. To remove the variability related to data size differences, all raw fastq data were in silico down-sampled to 11.5 M reads (35,000× raw read depth). First, we examined the sequencing metrics depending on the initial genomic DNA amounts (Fig. 3a, Additional file 7). As the amount of input genomic DNA decreased, the duplicate rate increased and the on-target rate decreased, which was similar to our previous results obtained by capture-based targeted sequencing [33]. However, the depth of unique coverage was higher than the previous result. By using the UMIs, a 3052× mean depth of unique coverage was achieved when 11.5 M sequencing reads were generated using 6.25 ng of genomic DNA as the input material. Second, we analyzed the depth of unique coverage depending on the generated data sizes and initial genomic DNA amounts (Fig. 3b). The depth of unique coverage increased as more raw data was generated, regardless of the input DNA amount. However, the rate of change in the unique coverage varied at a given interval of data size depending on the input DNA amounts. For example, when 50 ng was used, the increased trend of unique coverage depth was not dramatically attenuated at the data endpoint (35,000× coverage), indicating that the library complexity was significantly larger than the observed depth of unique coverage. In contrast, the increase curve was saturated at the same endpoint when 6.25 ng was used as an initial amount. Even if more data are generated over 35,000× using 6.25 ng, the target depth over 3000× would not considerably increase. Based on this result, it is possible to predict how much data will be obtained when only a small amount of genomic DNA is available for library construction. Third, we evaluated the detection sensitivity of the method using a reference sample that contained eight variants (six single nucleotide variants (SNVs) and two insertion and deletions (InDels)) at three different allelic fractions between 0.1–6.3% as described by the manufacturer (Additional file 8). We used the web-based Qiagen data analysis center to detect the variants (Additional file 9) by removal of duplicated reads with UMI and sequencing error corrections [34, 35]. When 25 or 50 ng was used for the library construction, all variants present at a frequency ≥ 1% were detected. However, two SNVs present at a 1% frequency were not detected when 12.5 or 6.25 ng was used. All variants (six SNVs and two InDels) present at a frequency less than 1% were not detected using any amount of initial genomic DNA. To compare the number of false positive calls, we first excluded 36 variant candidates including the eight SNVs listed by the manufacturer and 28 SNVs in the wild-type sample of the Horizon cfDNA reference set (HD776). More false positive variants were detected as the amount of input DNA decreased (Additional file 10). Furthermore, the number of false positive calls (VAF > 0.01) decreased after deduplication with the UMIs compared to that without the UMIs. In addition to the Qiagen web-based analysis, we detected variants with LoFreq and Pindel after filtering out duplicated reads with the UMIs. The result was not much different from the Qiagen web-based analysis as described in the discussion section (Additional file 9). We also examined the correlation between the expected allele frequency and the observed allele frequency of the detected variants according to the initial genomic DNA amount (Fig. 3c, Additional file 11). When a greater amount of input DNA was used, the correlation between the expected and observed allele frequencies was improved. These results indicated that a high depth of coverage increases the accuracy of predicting variant allele frequencies when large initial amounts are used. However, when using 6.25 ng as the starting amount, mutations present at a frequency as low as 1% could be detected. These results suggest that the efficient recovery of template molecules with this library construction kit resulted in the sensitive detection of low VAF variants.

## Discussion

Various target enrichment methods have been developed and improved for massively parallel sequencing, which has played a central role in allowing targeted deep sequencing to be a routine test for detecting alterations in cancer-associated genes in clinical laboratories. While each technique has advantages and disadvantages, the two most commonly used enrichment approaches are based on hybrid selection or highly multiplexed PCR. Hybrid selection-based target enrichment easily increases the number of targeted sites covering relatively broad genomic regions, but this method generally requires additional experimental procedures including hybridization for a relatively long time using synthesized oligonucleotide baits. The enrichment efficiency of PCR nearly always exceeds that of hybrid capture, but conventional PCR-based methods such as AmpliSeq, TruSeq Amplicon, and HaloPlex have no way of deduplication because of the identical position coordinates determined by each pair of PCR primers. Because deduplication helps to mitigate a potential skew in allele frequency estimation due to the inherent variability in the PCR amplification steps, the methods are more likely to generate false positives, particularly when calling low VAF variants. By introducing the UMI technology, the multiplex PCR-based enrichment technique may overcome the disadvantage of the absence of an efficient deduplication process, which clearly offers more choices for using targeted enrichment sequencing.

The sensitivity, specificity, accuracy, and precision of variant detection are tightly related to the mean depth of unique coverage. Because it correlates with molecular complexity and, in turn, the amount of input DNA, achieving a high depth of unique coverage is demanding, particularly when a small amount of DNA is used for library construction. In fact, tissue biopsies and plasma samples often produce only limited amounts of DNA to be used in analyzes. Thus, we evaluated the performance of commercial kits using a relatively small amount of DNA (50 ng or less) for the library construction. Furthermore, the mean depth of unique coverage may not be well correlated with library complexity, unless the majority of unique molecules in the libraries are sequenced. Sequencing the most unique molecules in a library might not be practical if the library is constructed with a large amount of DNA. The range of the initial amount of genomic DNA we tested was based on the situation of limited material or cfDNA collected from 5 mL of plasma.

Because the depth of unique coverage may be inflated due to errors in the UMIs, we estimated UMI errors using UMI-tools and subsequently adjusted the depth of coverage. All kits displayed minor inflation of the coverage depth because of errors in the UMIs. However, these adjusted coverage depths may be still somewhat inflated because UMI-tools deals with nucleotide substitutions but not InDels and chimeric sequences that arise from recombination events. Although they may not comprise a major fraction compared to substitution errors, InDels and chimeric errors in UMIs remain to be evaluated.

The method of tagging template molecules with UMIs differs among vendors. For the Nugen and NEB kits, it is limited to increasing the length of the UMI at the request of customers because the UMIs are placed next to the sample index. In contrast, the control of UMI length in the Qiagen and ArcherDx kits is relatively easier. However, the short sequence is essential to distinguish between the UMI and template to be sequenced, which forms a duplex region of y-shaped adaptors required for double-stranded DNA ligation. In addition, the usable read length is reduced because of the position of the UMI and duplex region. Basically, an increase in the coverage depth after deduplication using UMIs is related to the library complexity generated from each kit using the same initial amount of DNA. The difference of the library complexity might result from the effectiveness of each step of the library construction related to various elements including UMI position, length, and tagging process. While longer UMIs are more likely to accumulate errors, they may be necessary to generate greater complexity, particularly for higher sequencing depth experiments. As the optimal length of the UMIs may vary depending on the experimental conditions, such as input DNA amount, library construction efficiency, and sequencing data size, our study offers useful information for choosing the length of the UMIs.

Compared to our previous results obtained using a hybrid capture-based enrichment method, [33] the mean depths of unique coverage with the Qiagen kits considerably increased. The data indicated superior library construction efficiency, although the two methods were not compared in controlled experiments. This may occur partly because enrichment with the Qiagen kits targets both strands, while the capture-based enrichment method inevitably recovers only one strand. The difference in the library construction efficiency results between the two Qiagen kits might be due to the efficiency of the multiplex PCR with different numbers of primers and the primer design of each target region. More importantly, the targeted deep sequencing data generated from 6.25 ng of initial genomic DNA using the Qiagen kit resulted in sufficient depth of unique coverage for detecting variants present at an allele frequency as low as 1%. Because one diploid genome consists of 6.6 pg of DNA, 6.25 ng of gDNA is equivalent to 1982 haploid genomes. However, over 3000× unique coverage was generated using 6.25 ng of initial genomic DNA. Although it is theoretically possible to achieve 3964x by reading both strands, there might be some degree of overestimation due to overlaps between paired reads and/or errors in the UMI sequences. In this study, we attempted to detect SNVs/InDels using a web-based resource from the vendor as well as LoFreq [36] and Pindel [37]. When we used LoFreq and Pindel to detect variants without UMIs, the detection sensitivity was compromised. When using the UMIs, only one variant (1% AF of EGFR T790 M from 6.25 ng) was not detected in the 6.25 ng sample, but more variants were missed without using UMIs: 1% AF of EGFR L858R from 6.25 ng, 1.3% AF of NRAS Q61K from 12.5 and 25 ng, and 1.3% AF of PIK3CA E545K from 12.5 ng (Additional file 9). These results were reproduced in a duplicate experiment.

Because the detection sensitivity of low VAF variants also greatly depends on the variant calling algorithms, more sophisticated methods involving the suppression of background errors [27] may further improve the detection sensitivity of the assay. Nonetheless, our results revealed significant variability in several performance parameters among commercial library construction kits for PCR-based targeted sequencing. The Qiagen kit enabled the detection of variants present as low as 1%, even when less than 10 ng of genomic DNA was available.

In this study, most of the experiments for the comparisons were performed by using DNA extracted from cell lines, which are not a realistic substitute in many clinical situations. Testing the kits using clinical samples is needed for further evaluation. In addition, although we evaluated a number of the major available kits, the list of kits tested in this study is not exhaustive leaving a number of kits of this type not tested. In addition, more commercial PCR-based enrichment kits may become available as the technology evolves. Nevertheless, our study offers useful benchmark data for evaluating the performance of new kits for targeted deep sequencing.

## Conclusions

In this study, we compared commercial PCR-based target enrichment kits that include UMI sequences. The portion of duplicates dramatically decreased with the identification of unique molecules using UMIs in all kits. However, the kits varied in quality metrics such as library complexity (i.e., depth of unique coverage), coverage uniformity, and errors in the UMIs. Although no single kit outperformed the others in all aspects, the Qiagen HASTP kit displayed the highest library complexity and was chosen for further analytical performance evaluations. The targeted deep sequencing method based on PCR target enrichment combined with UMI tagging sensitively detected low VAF variants, even when using a limited amount of initial genomic DNA, suggesting the efficient recovery of template molecules. In summary, our results provide a practical guideline for researchers to choose appropriate options for PCR-based targeted sequencing. Furthermore, the data generated in this study would be useful benchmark data for evaluating new kits in the near future.

## Methods

### Cell lines and DNA

To compare the library construction efficiency, purified DNA from 10 normal HapMap cell lines (Additional file 12) were purchased from the Coriell Institute (http://ccr.coriell.org/). Genomic DNA was extracted from the cell lines using QIAamp DNA Mini Kits (Qiagen, Hilden, Germany). DNA concentration and purity were measured in a Picogreen fluorescence assay using a Qubit 2.0 Fluorometer (Life Technologies, Carlsbad, CA, USA) and a Nanodrop 8000 UV-Vis spectrometer (Thermo Scientific, Waltham, MA, USA). The fragment size distribution was measured using a 2200 TapeStation Instrument (Agilent Technologies, Santa Clara, CA, USA). To evaluate the performance of variant detection, the Horizon cfDNA reference standard set, HD777–800 (Horizon Discovery Group plc, Cambridge, UK), was used.

### Library preparation

A total of five library preparation kits were tested: the Archer® Reveal ctDNA™ 28 Kit (ArcherDX Inc., Boulder, CO, USA), NEBNext Direct® Cancer HotSpot Panel (New England Biolabs, Inc., Ipswich, MA, USA), Nugen Ovation® Custom Target Enrichment System (NuGEN Technologies, Inc., San Carlos, CA, USA), Qiagen Human Comprehensive Cancer Panel, and Qiagen Human Actionable Solid Tumor Panel. For the Nugen analysis, we used a custom-designed panel of 46.9 kb with the Ovation Target Enrichment System. When requesting the custom design, the use of short fragments such as cfDNA was considered as per the manufacturer’s description. Also, UMI sequences were included in that Nugen kit to distinguish PCR-duplicated fragments like the Ovation® Cancer Panel 2.0 Target Enrichment System. Thus, we thought that the Nugen custom kit should be sufficient to detect low allele frequency variants even though the low AF variant detection capability was not claimed on the specification sheet. Genomic DNA was fragmented to 150–200 bp by sonication using a Covaris S2 (7 min, 0.5% duty, intensity = 0.1, 50 cycles/burst; Covaris, Inc., Woburn, MA, USA) for three library preparation kits: the Archer® Reveal ctDNA™ 28, NEBNext Direct® Cancer HotSpot Panel, and Nugen Ovation® Custom Cancer Panel. In the Qiagen HCCP and HASTP, genomic DNA was fragmented in the fragmentation buffer included in the kits. After fragmentation of the genomic DNA, libraries were created according to the manufacturers’ protocols. To evaluate the performances of these kits, technically triplicated libraries were constructed and sequenced for each kit. In the assessment of the selected kit, the Qiagen HASTP, duplicate experiments were performed.

### Sequencing

The libraries were diluted to a final concentration of 2 nM and pooled by equal molarity. To sequence using the Hiseq 2500, all libraries were denatured by adding 0.2 nM NaOH and diluted to 20 pM with hybridization buffer from Illumina (San Diego, CA, USA). Sequencing was conducted according to the Hiseq 2500 instruction manual.

### Sequencing data process

Fastq files were aligned to the human reference sequence hg19 by BWA v0.7.5a [31] and sorted by SAMtools v0.1.18 [38]. Duplicated reads were marked by Picard v.1.93 without UMIs. For marking duplicated reads with UMIs, the fgbio package (https://github.com/fulcrumgenomics/fgbio) was used. Sequencing metrics were produced by Picard v1.93. To estimate and fix the error in the UMI sequence, UMI-tools was used. To accurately compare the kits, target regions in each kit were made by adding 100 bp from the end positions of the target-specific PCR primers, except for in the NEBnext direct® Cancer HotSpot Panel. Because the manufacturer policy did not reveal the genomic positions of the target-specific PCR primers, we used the target regions of the NEB kit indicated on their website. For the in silico down-sampling of the fastq files carried out by GATK v2.2 [39], we defined the raw read depth as the total number of bases divided by the total size (bp) of the target regions. For calling variants from the Qiagen HASTP, we used the Qiagen data analysis center and LoFreq and Pindel after the BAM files, in which duplicated reads were filtered out by UMIs. To calculate the allele fractions of non-detected variant positions, mpileup of SAMtools v0.1.18 was used.

## Additional files

Additional file 1: Characteristics of the five kits and QC metrics of the sequencing data. (XLSX 10 kb)

Additional file 2: Diagram of the library construction from four manufacturers. (JPG 124 kb)

Additional file 3: Summary of the methods to detect variants from the manufacturer. (XLSX 11 kb)

Additional file 4: Depth of unique coverage according to the size of the target regions (x-axis). Target regions adjacent to the 3′ ends of the gene-specific primers were expanded from 50 to 250 bp. (JPG 70 kb)

Additional file 5: Comparisons of uniformity across target regions. (a) The depth of each bin divided by the average depth of unique coverage was plotted across the entire target regions on a logarithmic scale. Red dashed lines are twice and half the average depth of coverage. Values (%) in red are the proportion of the target region out of the red line. (b) Coverage efficiency was visualized as the percentage of the total targeted bases covered at specific depths. (JPG 214 kb)

Additional file 6: Analysis of UMI errors. (a) The frequency of reads tagged with erroneous UMIs was estimated by UMI-tools. (b) Sequencing metrics obtained without UMIs, with UMIs, and with error-corrected UMIs using UMI-tools. The stacked bar plot shows the fractions of filtered reads (i.e., unaligned, duplicated, and off-target reads) and reads left after filtering (i.e., on-target) during raw data processing for five commercial kits with and without UMIs for deduplication. (JPG 149 kb)

Additional file 7: Sequencing metrics depending on input DNA amounts. Sequencing metrics were obtained without UMIs, with UMIs, and with error-corrected UMIs using UMI-tools. The stacked bar plot shows the fractions of filtered reads (i.e., unaligned, duplicated, and off-target reads) and reads left after filtering (i.e., on-target) during raw data processing for five commercial kits with and without UMIs for deduplication. (JPG 87 kb)

Additional file 8: Variants in Horizon cfDNA reference material. (XLSX 9 kb)

Additional file 9: Detection of variants with each detection method depending on various input DNA amounts. Success and failure of variant detection are indicated by ‘O’ and ‘X,’ respectively. AF, allele frequency; UMI, unique molecular identifier. (XLSX 10 kb)

Additional file 10: Summary of false negative calls when using various input DNA amounts. Listed positions are not detected in each condition. (XLSX 20 kb)

Additional file 11: Correlation between the expected allele frequency of variants in the reference material and observed allele frequency of variants obtained using the Qiagen HASTP. Because the variants present at allele frequencies of 0.1% or 0.13% were not detected by the Qiagen data analysis center or Lofreq/Pindel, the reads supporting the reference and alternative nucleotides at the corresponding positions were counted by mpielup to calculate the observed allele frequencies. (JPG 92 kb)

Additional file 12: List of HapMap cell lines used as the input DNA sources for performance evaluations of library construction kits. (XLSX 9 kb)

## Abbreviations

HASTP: Human Actionable Solid Tumor Panel
HCCP: Human Comprehensive Cancer Panel
UMI: Unique Molecular Identifier
VAF: Variant Allele Frequency
WES: Whole Exome Sequencing
WGS: Whole Genome Sequencing

## References

[1] Weinstein JN, Collisson EA, Mills GB, Shaw KR, Ozenberger BA, Ellrott K, Shmulevich I, Sander C, Stuart JM, Cancer Genome Atlas Research N (2013). The Cancer genome atlas pan-Cancer analysis project. Nat Genet.

[2] Hoadley KA, Yau C, Wolf DM, Cherniack AD, Tamborero D, Ng S, Leiserson MDM, Niu B, McLellan MD, Uzunangelov V (2014). Multiplatform analysis of 12 cancer types reveals molecular classification within and across tissues of origin. Cell.

[3] Cancer Genome Atlas Research N (2011). Integrated genomic analyses of ovarian carcinoma. Nature.

[4] Richards S, Aziz N, Bale S, Bick D, Das S, Gastier-Foster J, Grody WW, Hegde M, Lyon E, Spector E (2015). Standards and guidelines for the interpretation of sequence variants: a joint consensus recommendation of the American College of Medical Genetics and Genomics and the Association for Molecular Pathology. Genet Med.

[5] Forbes SA, Beare D, Boutselakis H, Bamford S, Bindal N, Tate J, Cole CG, Ward S, Dawson E, Ponting L (2017). COSMIC: somatic cancer genetics at high-resolution. Nucleic Acids Res.

[6] Mertes F, Elsharawy A, Sauer S, van Helvoort JM, van der Zaag PJ, Franke A, Nilsson M, Lehrach H, Brookes AJ (2011). Targeted enrichment of genomic DNA regions for next-generation sequencing. Brief Funct Genomics.

[7] Rohland N, Reich D (2012). Cost-effective, high-throughput DNA sequencing libraries for multiplexed target capture. Genome Res.

[8] Brunham LR, Hayden MR (2012). Medicine. Whole-genome sequencing: the new standard of care?. Science.

[9] Iglesias A, Anyane-Yeboa K, Wynn J, Wilson A, Truitt Cho M, Guzman E, Sisson R, Egan C, Chung WK (2014). The usefulness of whole-exome sequencing in routine clinical practice. Genet Med.

[10] Sausen M, Phallen J, Adleff V, Jones S, Leary RJ, Barrett MT, Anagnostou V, Parpart-Li S, Murphy D, Kay Li Q (2015). Clinical implications of genomic alterations in the tumour and circulation of pancreatic cancer patients. Nat Commun.

[11] Xuan J, Yu Y, Qing T, Guo L, Shi L (2013). Next-generation sequencing in the clinic: promises and challenges. Cancer Lett.

[12] Sims D, Sudbery I, Ilott NE, Heger A, Ponting CP (2014). Sequencing depth and coverage: key considerations in genomic analyses. Nat Rev Genet.

[13] Meynert AM, Ansari M, FitzPatrick DR, Taylor MS (2014). Variant detection sensitivity and biases in whole genome and exome sequencing. BMC Bioinformatics.

[14] Shin HT, Choi YL, Yun JW, Kim NKD, Kim SY, Jeon HJ, Nam JY, Lee C, Ryu D, Kim SC (2017). Prevalence and detection of low-allele-fraction variants in clinical cancer samples. Nat Commun.

[15] Hata AN, Niederst MJ, Archibald HL, Gomez-Caraballo M, Siddiqui FM, Mulvey HE, Maruvka YE, Ji F, Bhang HE, Krishnamurthy Radhakrishna V (2016). Tumor cells can follow distinct evolutionary paths to become resistant to epidermal growth factor receptor inhibition. Nat Med.

[16] Al-Kateb H, Nguyen TT, Steger-May K, Pfeifer JD (2015). Identification of major factors associated with failed clinical molecular oncology testing performed by next generation sequencing (NGS). Mol Oncol.

[17] Murtaza M, Dawson SJ, Tsui DW, Gale D, Forshew T, Piskorz AM, Parkinson C, Chin SF, Kingsbury Z, Wong AS (2013). Non-invasive analysis of acquired resistance to cancer therapy by sequencing of plasma DNA. Nature.

[18] Newman AM, Bratman SV, Wynne JF, Eclov NC, Modlin LA, Liu CL, Neal JW, Wakelee HA, Merritt RE, To J (2014). An ultrasensitive method for quantitating circulating tumor DNA with broad patient coverage. Nat Med.

[19] Volik S, Alcaide M, Morin RD, Collins C (2016). Cell-free DNA (cfDNA): clinical significance and utility in Cancer shaped by emerging technologies. Mol Cancer Res.

[20] Bettegowda C, Sausen M, Leary RJ, Kinde I, Wang Y, Agrawal N, Bartlett BR, Wang H, Luber B, Alani RM (2014). Detection of circulating tumor DNA in early- and late-stage human malignancies. Sci Transl Med.

[21] Gnirke A, Melnikov A, Maguire J, Rogov P, LeProust EM, Brockman W, Fennell T, Giannoukos G, Fisher S, Russ C (2009). Solution hybrid selection with ultra-long oligonucleotides for massively parallel targeted sequencing. Nat Biotechnol.

[22] Summerer D (2009). Enabling technologies of genomic-scale sequence enrichment for targeted high-throughput sequencing. Genomics.

[23] Kivioja T, Vaharautio A, Karlsson K, Bonke M, Enge M, Linnarsson S, Taipale J (2011). Counting absolute numbers of molecules using unique molecular identifiers. Nat Methods.

[24] Schmitt MW, Kennedy SR, Salk JJ, Fox EJ, Hiatt JB, Loeb LA (2012). Detection of ultra-rare mutations by next-generation sequencing. Proc Natl Acad Sci U S A.

[25] Kinde I, Wu J, Papadopoulos N, Kinzler KW, Vogelstein B (2011). Detection and quantification of rare mutations with massively parallel sequencing. Proc Natl Acad Sci U S A.

[26] Islam S, Zeisel A, Joost S, La Manno G, Zajac P, Kasper M, Lonnerberg P, Linnarsson S (2014). Quantitative single-cell RNA-seq with unique molecular identifiers. Nat Methods.

[27] Newman AM, Lovejoy AF, Klass DM, Kurtz DM, Chabon JJ, Scherer F, Stehr H, Liu CL, Bratman SV, Say C (2016). Integrated digital error suppression for improved detection of circulating tumor DNA. Nat Biotechnol.

[28] Phallen J, Sausen M, Adleff V, Leal A, Hruban C, White J, Anagnostou V, Fiksel J, Cristiano S, Papp E (2017). Direct detection of early-stage cancers using circulating tumor DNA. Sci Transl Med.

[29] Scolnick JA, Dimon M, Wang IC, Huelga SC, Amorese DA (2015). An efficient method for identifying gene fusions by targeted RNA sequencing from fresh frozen and FFPE samples. PLoS One.

[30] Zheng Z, Liebers M, Zhelyazkova B, Cao Y, Panditi D, Lynch KD, Chen J, Robinson HE, Shim HS, Chmielecki J (2014). Anchored multiplex PCR for targeted next-generation sequencing. Nat Med.

[31] Li H, Durbin R (2010). Fast and accurate long-read alignment with burrows-wheeler transform. Bioinformatics.

[32] Smith T, Heger A, Sudbery I (2017). UMI-tools: modeling sequencing errors in unique molecular identifiers to improve quantification accuracy. Genome Res.

[33] Chung J, Son DS, Jeon HJ, Kim KM, Park G, Ryu GH, Park WY, Park D (2016). The minimal amount of starting DNA for Agilent's hybrid capture-based targeted massively parallel sequencing. Sci Rep.

[34] Data Analysis Center [https://www.qiagen.com/kr/shop/genes-and-pathways/data-analysis-center-overview-page/].

[35] Xu C, Nezami Ranjbar MR, Wu Z, DiCarlo J, Wang Y (2017). Detecting very low allele fraction variants using targeted DNA sequencing and a novel molecular barcode-aware variant caller. BMC Genomics.

[36] Wilm A, Aw PP, Bertrand D, Yeo GH, Ong SH, Wong CH, Khor CC, Petric R, Hibberd ML, Nagarajan N (2012). LoFreq: a sequence-quality aware, ultra-sensitive variant caller for uncovering cell-population heterogeneity from high-throughput sequencing datasets. Nucleic Acids Res.

[37] Ye K, Schulz MH, Long Q, Apweiler R, Ning Z (2009). Pindel: a pattern growth approach to detect break points of large deletions and medium sized insertions from paired-end short reads. Bioinformatics.

[38] Li H, Handsaker B, Wysoker A, Fennell T, Ruan J, Homer N, Marth G, Abecasis G, Durbin R (2009). Genome project data processing S: the sequence alignment/map format and SAMtools. Bioinformatics.

[39] McKenna A, Hanna M, Banks E, Sivachenko A, Cibulskis K, Kernytsky A, Garimella K, Altshuler D, Gabriel S, Daly M (2010). The genome analysis toolkit: a MapReduce framework for analyzing next-generation DNA sequencing data. Genome Res.

