# Supplementary figures and images for: Performance evaluation of commercial library construction kits for PCR-based targeted sequencing using a unique molecular identifier
Source: BMC Genomics. 2019 Mar 14;20:216. doi: 10.1186/s12864-019-5583-7 (PMC6416880; doi:10.1186/s12864-019-5583-7)

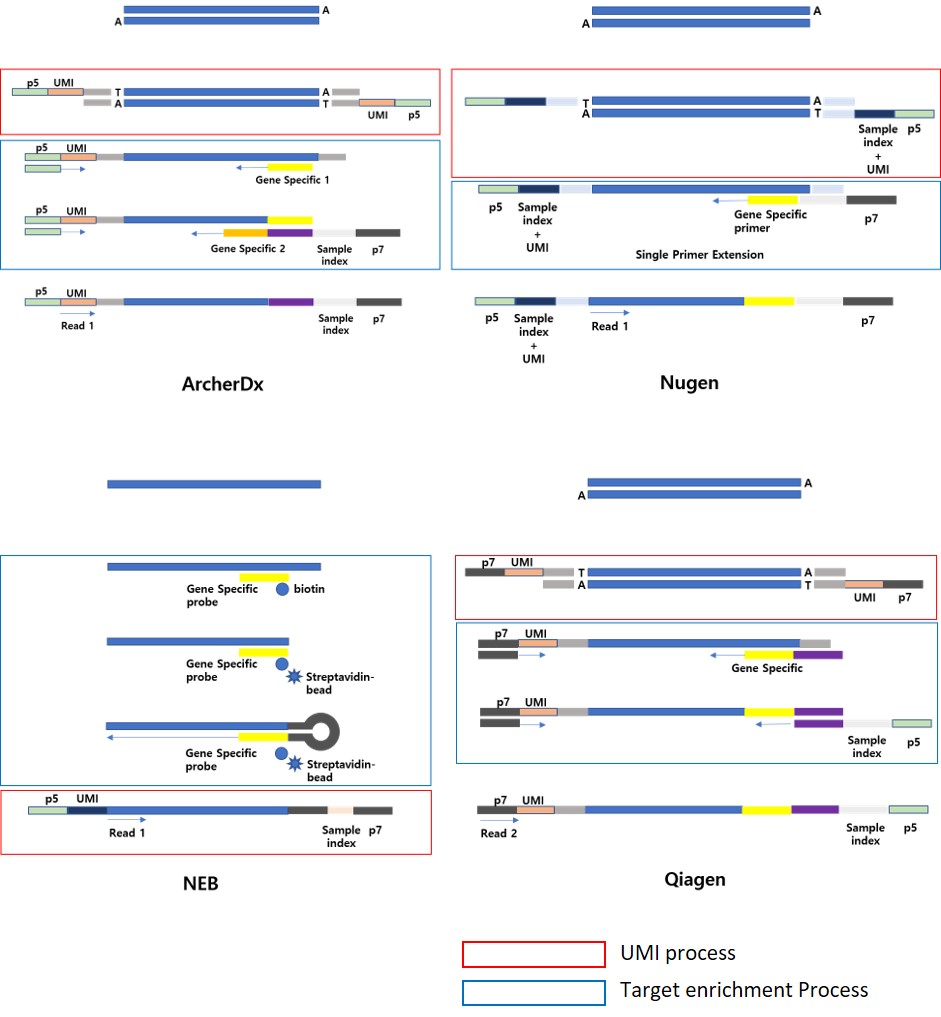

Supplement: Supplementary file 2 — Diagram of the library construction from four manufacturers. (JPG 124 kb) [file 12864_2019_5583_MOESM2_ESM.jpg]

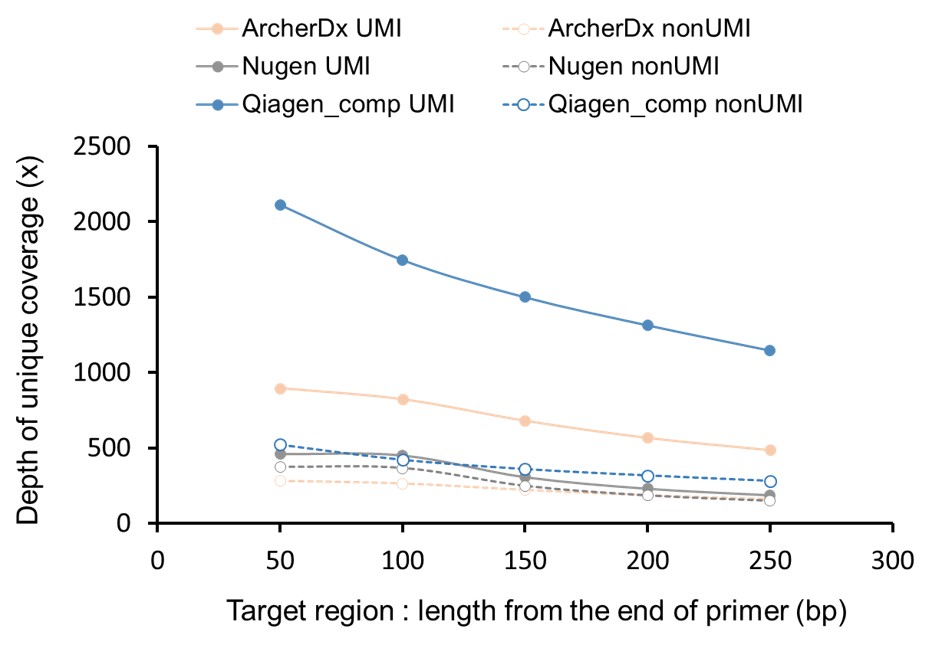

Supplement: Supplementary file 4 — Depth of unique coverage according to the size of the target regions (x-axis). Target regions adjacent to the 3′ ends of the gene-specific primers were expanded from 50 to 250 bp. (JPG 70 kb) [file 12864_2019_5583_MOESM4_ESM.jpg]

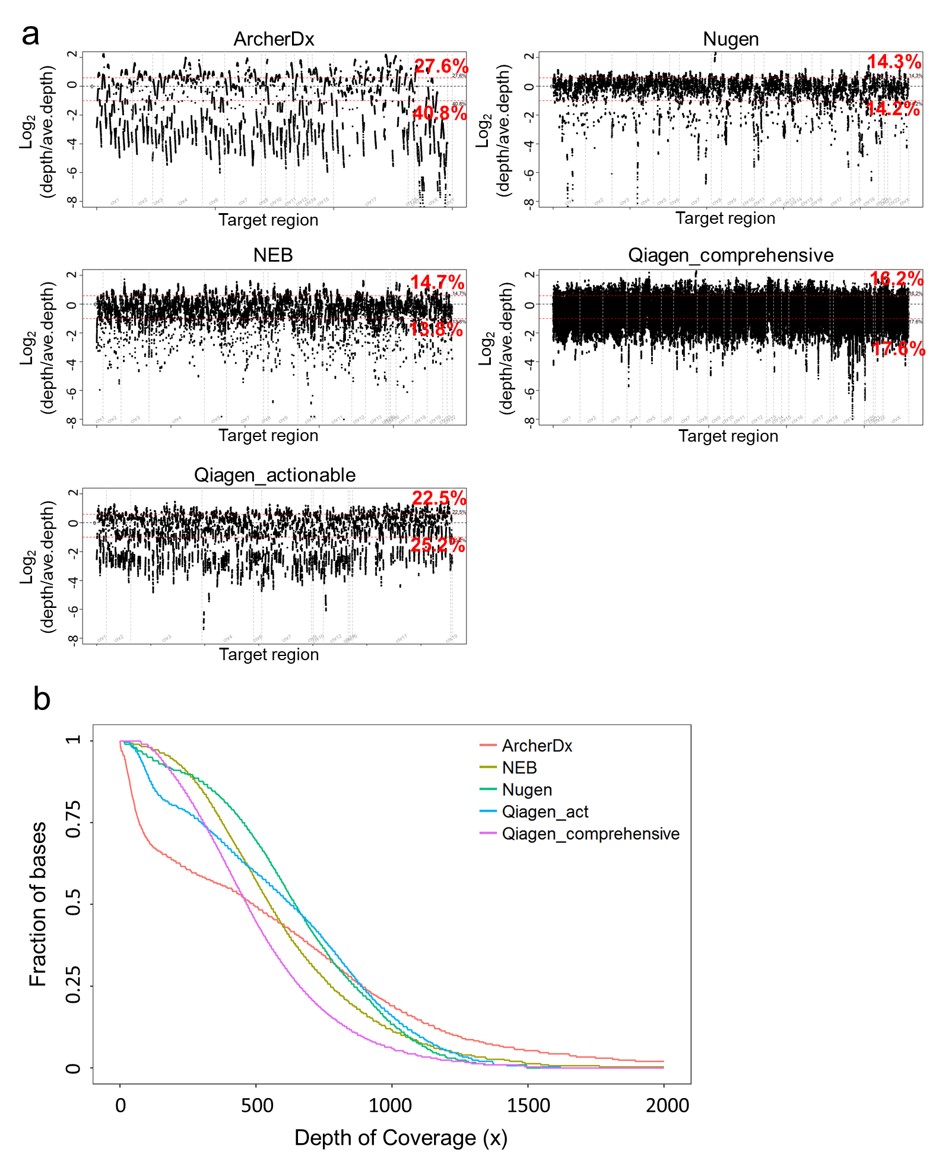

Supplement: Supplementary file 5 — Comparisons of uniformity across target regions. (a) The depth of each bin divided by the average depth of unique coverage was plotted across the entire target regions on a logarithmic scale. Red dashed lines are twice and half the average depth of coverage. Values (%) in red are the proportion of the target region out of the red line. (b) Coverage efficiency was visualized as the percentage of the total targeted bases covered at specific depths. (JPG 214 kb) [file 12864_2019_5583_MOESM5_ESM.jpg]

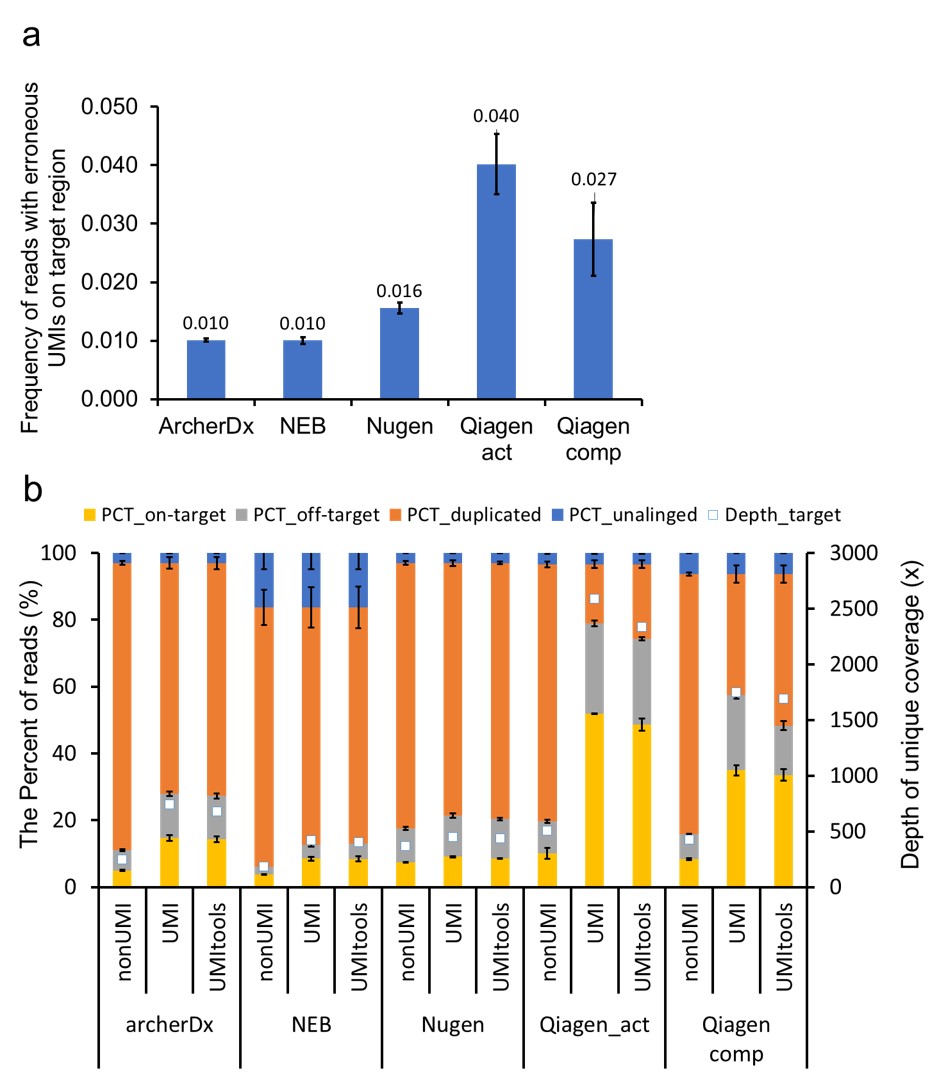

Supplement: Supplementary file 6 — Analysis of UMI errors. (a) The frequency of reads tagged with erroneous UMIs was estimated by UMI-tools. (b) Sequencing metrics obtained without UMIs, with UMIs, and with error-corrected UMIs using UMI-tools. The stacked bar plot shows the fractions of filtered reads (i.e., unaligned, duplicated, and off-target reads) and reads left after filtering (i.e., on-target) during raw data processing for five commercial kits with and without UMIs for deduplication. (JPG 149 kb) [file 12864_2019_5583_MOESM6_ESM.jpg]

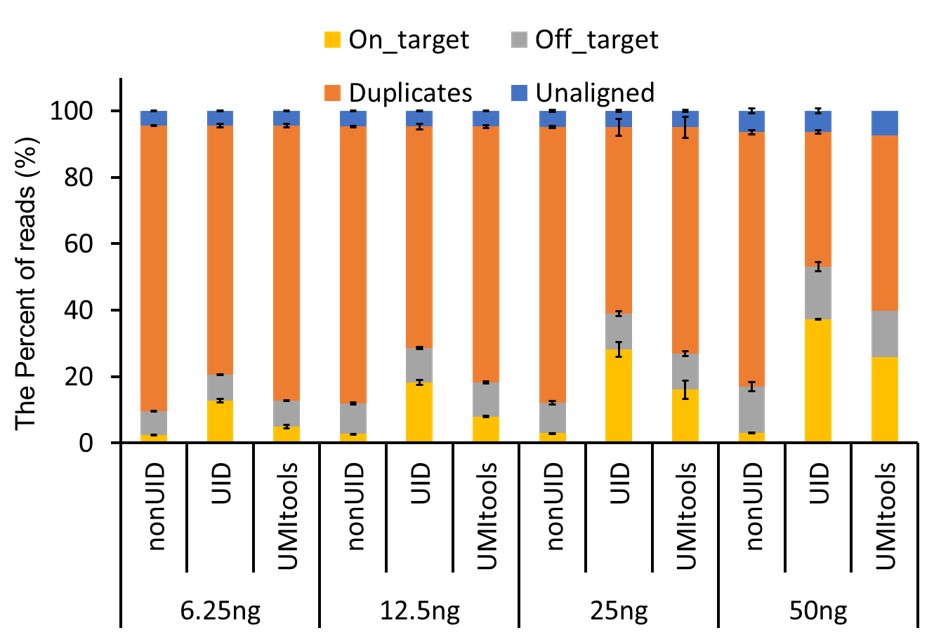

Supplement: Supplementary file 7 — Sequencing metrics depending on input DNA amounts. Sequencing metrics were obtained without UMIs, with UMIs, and with error-corrected UMIs using UMI-tools. The stacked bar plot shows the fractions of filtered reads (i.e., unaligned, duplicated, and off-target reads) and reads left after filtering (i.e., on-target) during raw data processing for five commercial kits with and without UMIs for deduplication. (JPG 87 kb) [file 12864_2019_5583_MOESM7_ESM.jpg]

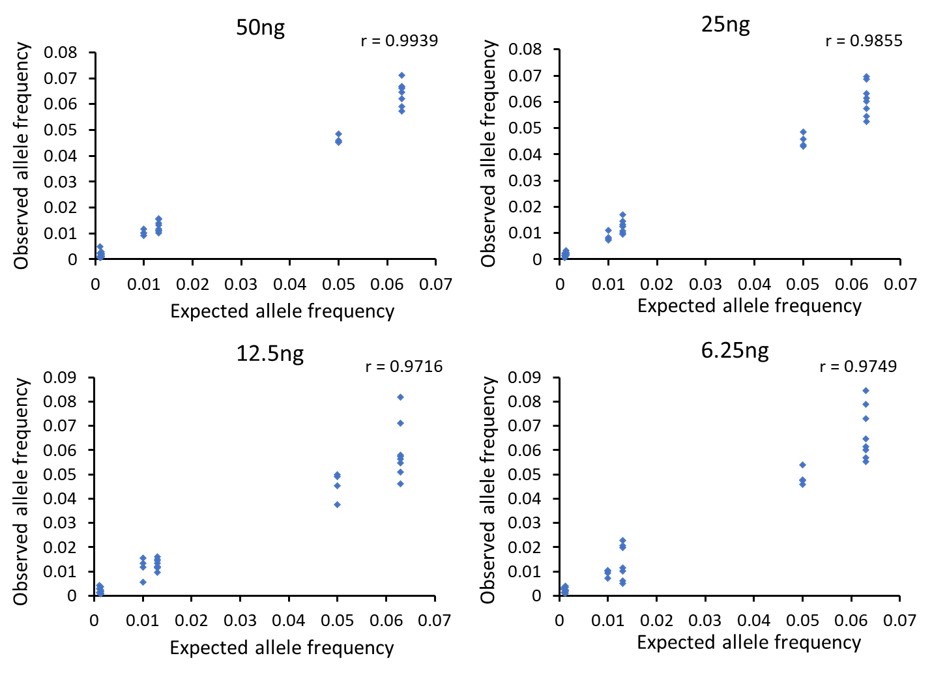

Supplement: Supplementary file 11 — Correlation between the expected allele frequency of variants in the reference material and observed allele frequency of variants obtained using the Qiagen HASTP. Because the variants present at allele frequencies of 0.1% or 0.13% were not detected by the Qiagen data analysis center or Lofreq/Pindel, the reads supporting the reference and alternative nucleotides at the corresponding positions were counted by mpielup to calculate the observed allele frequencies. (JPG 92 kb) [file 12864_2019_5583_MOESM11_ESM.jpg]
